# Combination of palbociclib with enzalutamide shows *in vitro* activity in RB proficient and androgen receptor positive triple negative breast cancer cells

**DOI:** 10.1371/journal.pone.0189007

**Published:** 2017-12-20

**Authors:** Chun-Yu Liu, Ka-Yi Lau, Chia-Chi Hsu, Ji-Lin Chen, Chia-Han Lee, Tzu-Ting Huang, Yi-Ting Chen, Chun-Teng Huang, Po-Han Lin, Ling-Ming Tseng

**Affiliations:** 1 Comprehensive Breast Health Center, Taipei Veterans General Hospital, Taipei, Taiwan; 2 School of Medicine, National Yang-Ming University, Taipei, Taiwan; 3 Division of Medical Oncology, Department of Oncology, Taipei Veterans General Hospital, Taipei, Taiwan; 4 Division of Hematology & Oncology, Department of Medicine, Yang-Ming Branch of Taipei City Hospital, Taipei, Taiwan; 5 Department of Medical Genetics, National Taiwan University Hospital, Taipei, Taiwan; 6 Department of Surgery, Taipei Veterans General Hospital, Taipei, Taiwan; University of Wisconsin Madison, UNITED STATES

## Abstract

**Objectives:**

Triple negative breast cancer (TNBC) lacks specific drug targets and remains challenging. Palbociclib, a cyclin-dependent kinases 4 and 6 (CDK4/6) inhibitor is approved for metastatic estrogen receptor (ER)-positive and human epithermal growth factor 2 (HER2)-negative breast cancer. The nature of cell cycle inhibition by palbociclib suggests its potential in TNBC cells. Retinoblastoma (RB, a known substrate of CDK4/6) pathway deregulation is a frequent occurrence in TNBC and studies have revealed that pharmacological CDK4/6 inhibition induces a cooperative cytostatic effect with doxorubicin in RB-proficient TNBC models. In addition, recent studies reported that anti-androgen therapy shows preclinical efficacy in androgen-receptor (AR)-positive TNBC cells. Here we examined the effect of palbociclib in combination with an anti-androgen enzalutamide in TNBC cells.

**Method:**

MDA-MB-453, BT-549, MDA-MB-231 and MDA-MB-468 TNBC cell lines were used for *in vitro* studies. Protein expressions were assessed by Western blot analysis. Cytostatic effect was examined by MTT assay. Cell cycle and apoptosis were examined by flow cytometry.

**Results:**

Palbociclib showed inhibitory effect in RB-proficient TNBC cells, and enzalutamide inhibited cell viability in AR-positive TNBC cells. Enzalutamide treatment could enhance the palbociclib-induced cytostatic effect in AR-positive/RB-proficient TNBC cells. In addition, palbociclib-mediated G1 arrest in AR-positive/RB-proficient TNBC cells was attenuated by RB knockdown.

**Conclusion:**

Our study provided a preclinical rationale in selecting patients who might have therapeutic benefit from combining CDK4/6 inhibitors with AR antagonists.

## Introduction

Triple-negative breast cancer (TNBC) remains a challenging breast cancer subtype for its higher risk of distant recurrence, and poorer outcome after recurrence or metastasis than other types of breast cancer [1–3]. Targeted therapy for TNBC is emerging in clinical trials and recent molecular profiling studies have revealed molecular heterogeneity of TNBC [4], highlighting the importance of finding biomarkers for targeted therapy guidance for TNBC.

Palbociclib is a highly selective cyclin-dependent kinases 4 and 6 (CDK4/6) inhibitor, which blocks the phosphorylation of retinoblastoma protein (pRB) and subsequently arrests cell cycle at G1-phase [5, 6]. Previous study *in vitro* showed that palbociclib in combination with hormone therapy (tamoxifen) or target therapy (trastuzumab) had an effectively inhibitory effect on ER-positive and HER2-amplified breast cancer, respectively [7]. In clinical, palbociclib in combination with letorzole (aromatase inhibitor) has been approved by the U.S. Food and Drug Administration (FDA) for the treatment of patients with ER-positive and HER2-negative advanced breast cancer [8, 9]. However, the effects of palbociclib in TNBC are not well-documented.

Enzalutamide, an androgen receptor antagonist, has been approved by the FDA for the treatment of patients with metastatic prostate cancer [10, 11]. Cumulative evidences showed that enzalutamide has potent anti-tumor effects on TNBC cells, and suggested that androgen receptor (AR) might be a promising target for treatment of TNBC [12–14]. However, the effect of combination palbociclib with enzalutamide in TNBC cells is still unclear.

In present study, we tested the combination effect of palbociclib with enzalutamide in TNBC cells. Cytostatic effects of enzalutamide, palbociclib or combined treatment and effects of treatments on AR and pRB proteins expressions were examined. Moreover, the influences on cell cycle distribution and apoptosis were also evaluated.

## Materials and methods

### Cell culture and transfection

Human TNBC cell lines MDA-MB-453, MDA-MB-231, MDA-MB-468, BT-20 and HCC1937 cells and human breast epithelial cell line MCF 10A cells were cultured in Dulbecco’s Modified Eagle Medium (DMEM). Human TNBC cell lines BT-549 cells were cultured in RPMI 1640 medium with 0.023 UI/ml insulin. The complete growth medium was supplemented with 10% FBS, 0.1 mM non-essential amino acids, 2 mM L-glutamine, 100 units/mL penicillin G, 100 μg/mL streptomycin sulphate and 25 μg/mL amphotericin B in 37°C humidified incubator and an atmosphere of 5% CO_2_ in air. Cell lines were purchased from American Type Culture Collection (Manassas, VA, USA). For transfection, cells were seeded into 6-well for 24 h and transiently transfected by Lipofectamine 3000 Reagent (Thermo Fisher Scientific). For knockdown validation, ON-TARGETplus Human RB1 siRNA (GE Healthcare Dharmacon, E-003296-00-0005) was used to knockdown the endogenous RB1, and ON-TARGETplus Non-targeting Pool (GE Healthcare Dharmacon, D-001810-10-20) as a negative control.

### Western blot analysis

Whole cell extracts were prepared using RIPA buffer (Thermo Scientific) with a Halt Protease and Phosphatase Inhibitor Cocktail (Thermo Scientific). The protein concentrations were determined using the Bradford assay (Sigma-Aldrich). Samples were diluted in 5X Laemmli buffer (300 mM Tris-HCl pH 6.8, 10% SDS (w/v), 5% 2-mercaptoethanol, 25% glycerol (v/v), 0.1% bromphenol blue w/v) and boiled for 5 min. 35 μg of proteins were separated by 8–15% SDS-PAGE and transferred onto polyvinylidene fluoride (PVDF) membranes (PALL Life Science). Unspecific binding sites on the PVDF membranes were blocked with 5% non-fat milk in TBST (20 mM Tris-HCl, pH 7.6, 137 mM NaCl, 1% Tween-20). Membranes were hybridized with antibodies against anti-AR (Santa Cruz), anti-CDKN2A/p16INK4α (Abcam), anti-RB, anti-phospho-Rb (Ser780), anti-p21 Waf1/Cip1, anti-cyclin D1 and anti-β-actin (Cell Signaling Technology) for overnight at 4°C, followed by incubation with horseradish peroxidase (HRP)-conjugated secondary antibodies for 1 h at room temperature. The membranes were then developed using Immobilon Western chemiluminescence HRP substrates (Millipore). Images were captured by Luminescence/Fluorescence Imaging System (GE Healthcare).

### MTT (3-[4,5-Dimethylthiazol-2-y1]-2,5-diphenyltetrazolium bromide) assay

Cells were seeded into 96-well plates allowed to attach for 24 h and treated with indicated concentrations of enzalutamide or palbociclib for 72 h. The treated cells were added 0.5 mg/mL MTT (Sigma-Aldrich) to each well and incubated for 4 h at 37°C. The violet MTT formazan precipitates were subsequently dissolved in 100 μL DMSO. The absorbance at 570 nm was measured on an UQuant reader.

### Cell cycle analysis

The cell cycle assay was carried out by flow cytometry. Cells were collected and fixed with 70% chilled ethanol for overnight. Then, the cells were stained with 0.2 mg/mL Propidium Iodide (PI) for 30 min in the presence of 100 μg/mL RNase and protected from light. The stained cells were suspended in 500 μL phosphate buffered saline (PBS) and analyzed by flow cytometry. Data was gated and analyzed by the CellQuest software.

### Flow cytometry analysis for apoptosis detection

The apoptotic cells were measured by APC Annexin V (BD Biosciences) and PI double staining assay. Cells were seeded onto 6-well plates for overnight and then treated with enzalutamide or palbociclib for 48 h. The treated cells were harvested and suspended 1 x 10^6^ cells in 1 mL 1X Binding Buffer (BD Biosciences). 5 μL APC Annexin V and 4 μL PI were added into each sample and mixed. Cells were incubated for 15 min at room temperature prevented from light and then analyzed by flow cytometry.

## Results

### Effect of palbociclib and enzalutamide on AR and RB protein expressions in TNBC cell lines

**Fig 1:**
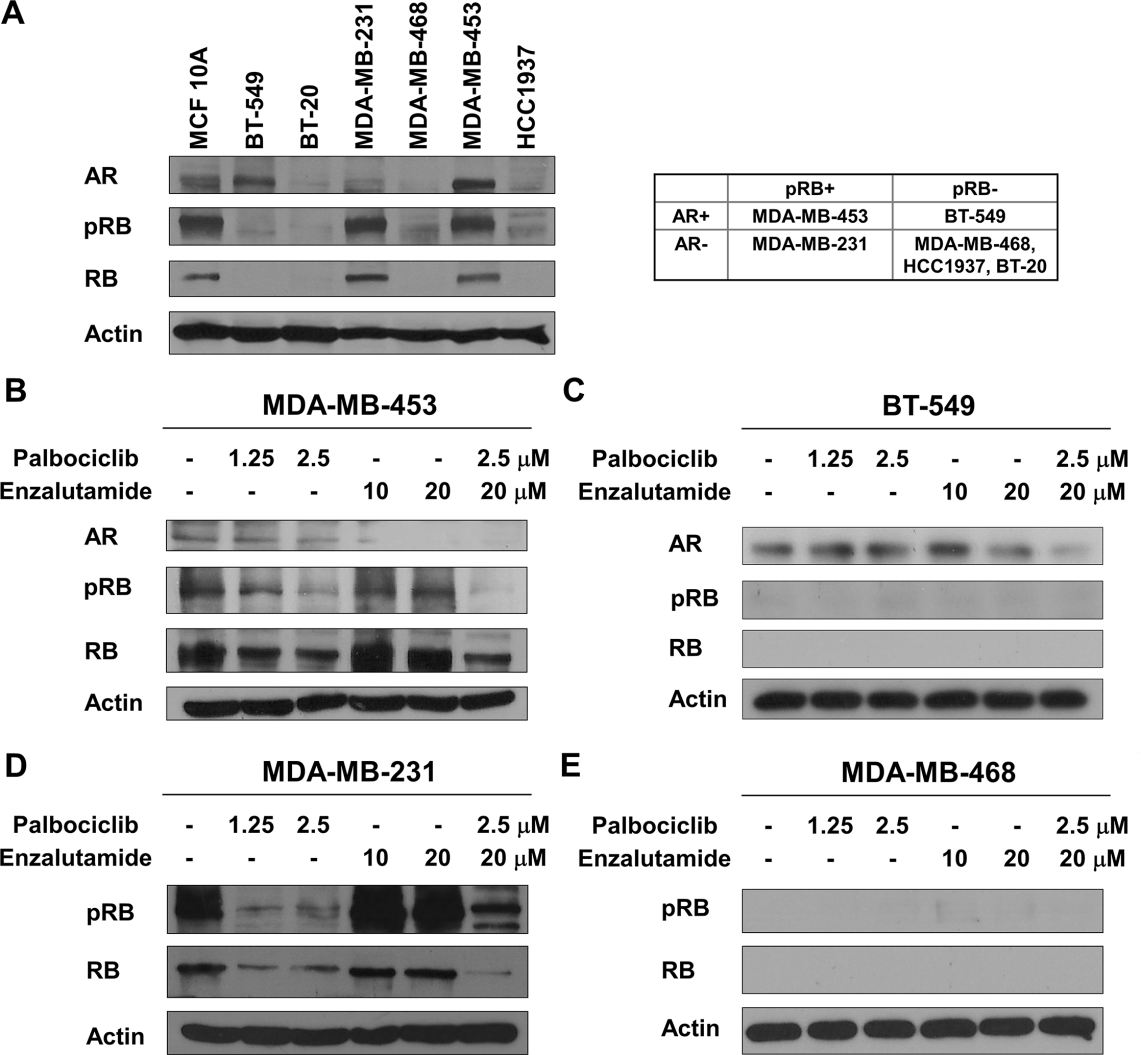
Effect of palbociclib and enzalutamide on AR and RB protein expressions in TNBC cell lines. (A) The whole cell extracts of MCF 10A, BT-549, BT-20 MDA-MB-231, MDA-MB-453, MDA-MB-468 and HCC1937 cells were analyzed by Western blot using antibodies against anti-AR, anti-phospho-RB, anti-RB and anti-β-actin. (B-D) The whole cell extracts of MDA-MB-453, BT-549, MDA-MB-231 and MDA-MB-468 were treated with DMSO (-), 1.25 and 2.5 μM palbociclib, 10 and 20 μM enzalutamide and combination of 2.5 μM palbociclib and 20 μM enzalutamide for 48 h which prepared for Western blot analysis using antibodies against anti-AR, anti-phospho-RB, anti-RB and anti-β-actin.

To develop precision medicine for TNBC subtypes, we first examined endogenous AR and RB expressions in TNBC cell lines and human breast epithelial cell line MCF 10A cells. The result showed that MDA-MB-453 and BT-549 is AR-positive cell lines, and RB and phosphorylated RB could be detected in MDA-MB-453 and MDA-MB-231 (Fig 1A). Moreover, we also confirmed that enzalutamide and palbociclib were effective against AR and RB protein expression, respectively (Fig 1B–1E). These results indicated that these TNBC cell lines could be classified into four categories, including AR-positive/RB-proficient (MDA-MB-453), AR-positive/RB-negative (BT-549), AR-negative/RB-proficient (MDA-MB-231) and AR-negative/RB-negative (MDA-MB-468, HCC1937 and BT-20), respectively.

### Enzalutamide in combination with palbociclib enhances the cytostatic effect in AR-positive/RB-proficient TNBC cells

**Fig 2:**
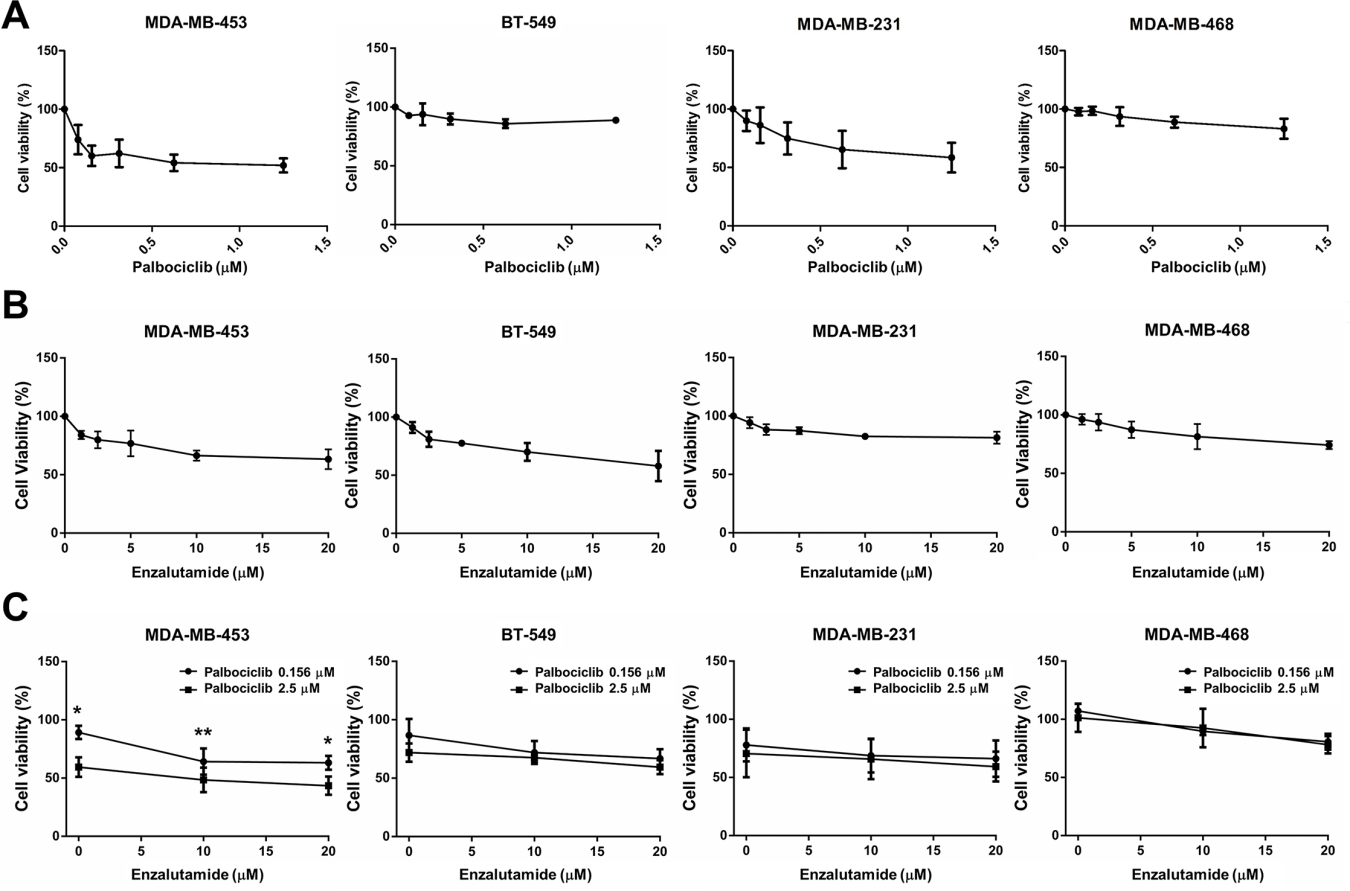
Enzalutamide in combination with palbociclib enhances the cytostatic effect in AR-positive/RB-proficient TNBC cells. (A) MDA-MB-453, BT-549, MDA-MB-231 and MDA-MB-468 cells were treated with various concentrations of palbociclib, (B) enzalutamide and (C) combination of 0.156 or 2.5 μM palbociclib and 10 or 20 μM enzalutamide for 72 h, the cell viability was determined using MTT assay. The means ± SEM of three independent experiments performed in triplicate are shown. *P < 0.05; **P < 0.01.

Since palbociclib is a selective CDK4/6 inhibitor, which blocks the phosphorylation of RB and subsequently leads to inhibition of cell growth, we evaluated the effect of palbociclib in three TNBC cell lines. The result showed that palbociclib significantly inhibited the cell growth in RB-proficient cells (MDA-MB-453 and MDA-MB-231) but not in RB-negative cells (MDA-MB-468) (Fig 2A). Enzalutamide-suppressed cell viability of AR-positive cells is nearly 50% (Fig 2B). To examine whether enzalutamide enhanced palbociclib-induced cytostatic effect, we used 0.156 or 2.5 μM palbociclib combined with various concentrations of enzalutamide in TNBC cell lines. The results revealed that enzalutamide enhanced the palbociclib-induced cytostatic effect in AR-positive/RB-proficient (MDA-MB-453) cells rather than AR-positive/RB-negative (BT-549), AR-negative/RB-proficient (MDA-MB-231), AR-negative/RB-negative (MDA-MB-468) cells (Fig 2C) and human breast epithelial cell line MCF 10A cells (S1 Fig). These results suggested that AR and RB protein expression might be important for the response to combined treatment with palbociclib and enzalutamide in TNBC cells.

### Presence of AR and RB enhance palbociclib-induced G1 arrest in TNBC cells

**Fig 3:**
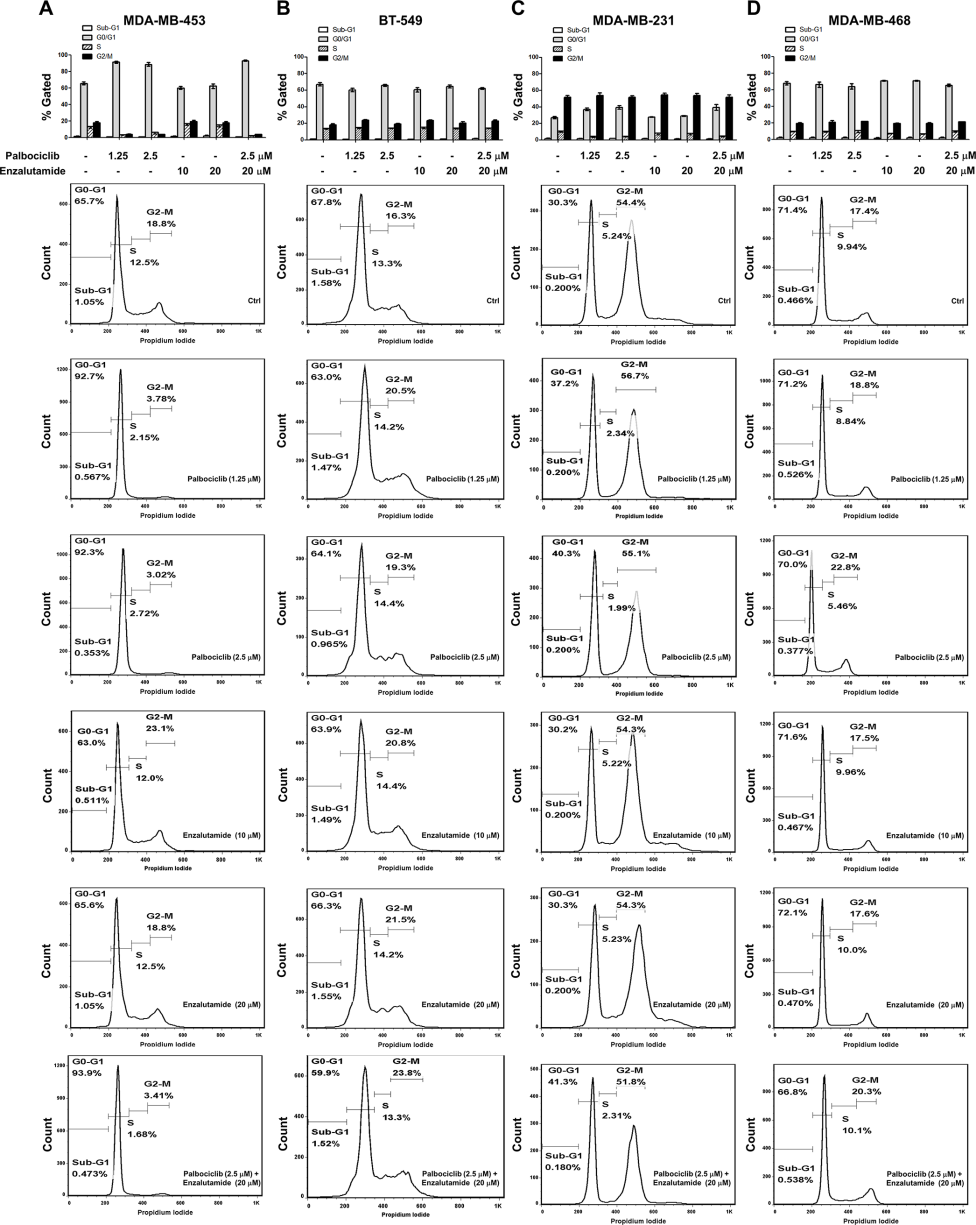
Palbociclib-induced G1 arrest is increased in AR-positive/RB-proficient cells. (A) MDA-MB-453, (B) BT-549, (C) MDA-MB-231 and (D) MDA-MB-468 cells were treated with DMSO (-), 1.25 and 2.5 μM palbociclib, 10 and 20 μM enzalutamide and combination of 2.5 μM palbociclib and 20 μM enzalutamide for 48 h, the cell cycle analysis by DNA content using flow cytometry. The means ± SEM of three independent experiments performed in triplicate are shown.

It is known that palbociclib decreases the phosphorylation of RB by inhibiting CDK4/6 and subsequently promotes cell cycle arrest at G1 phase, we next examined whether combined treatment of enzalutamide would alter the pattern of cell cycle in the presence of palbociclib. Enzalutamide treatment did not alter cell cycle distribution in TNBC cell lines. Palbociclib treatment slightly increased the cells in G1 phase of AR-negative/RB-proficient (MDA-MB-231) cells. However, palbociclib treatment significantly induced G1 arrest in AR-positive/RB-proficient (MDA-MB-453) cells but not in AR-positive/RB-negative (BT-549) and AR-negative/RB-negative (MDA-MB-468) cells (Fig 3A–3D). Palbociclib-induced G1 phase is nearly plateau (~90%) in MDA-MB-453 cells which might partly interpret that enzalutamide treatment did not further enhance palbociclib-induced G1 arrest in MDA-MB-453 cells.

**Fig 4:**
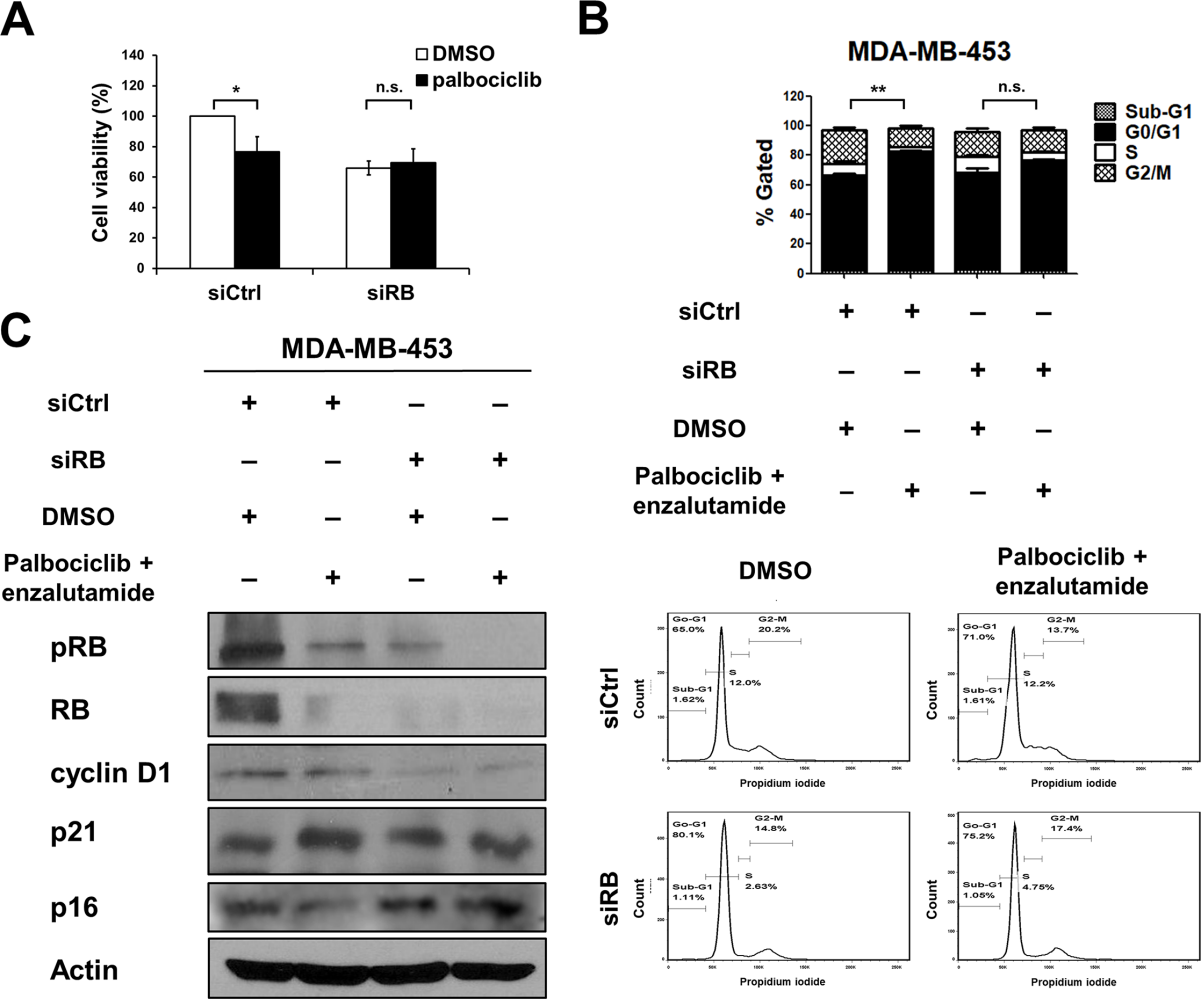
The effects of combination of palbociclib and enzalutamide are attenuated by RB knockdown. (A) MDA-MB-453 cells were transfected with siRNA against control (siCtrl) or RB1 (siRB) for 24 h, and the transfected cells were further treated with 2.5 μM palbociclib for 48 h. The treated cells were analyzed by MTT assay. *P < 0.05. (B, C) MDA-MB-453 cells were transfected with siRNA against control (siCtrl) or RB1 (siRB) for 24 h, and the transfected cells were further treated with 2.5 μM palbociclib and 20 μM enzalutamide for 48 h. The treated cells were analyzed by flow cytometry analysis (B) and Western blot (C) using antibodies against anti-phospho-RB, anti-RB, anti-cyclin D1, anti-p21, anti-p16 and anti-β-actin. G0/G1 subpopulation; **P < 0.01.

To explore whether concurrent expression of AR and RB was involved in palbociclib-induced G1 arrest, we analyzed the effects of palbociclib treatment in RB-knockdown MDA-MB-453 cells. Palbociclib treatment reduced cell viability in MDA-MB-453 cells. Knockdown of RB1 attenuated palbociclib-suppressed cell viability (Fig 4A). Palbociclib-induced G1 arrest was also attenuated by RB knockdown (S2 Fig). We further analyzed the effects of combination of palbociclib and enzalutamide in cell cycle regulation. Results showed G1 arrest was attenuated by RB knockdown (Fig 4B) Treatment both of palbociclib and enzalutamide upregulated p21 and downregulated p16 whereas the effects were reduced by RB knockdown (Fig 4C). These results might suggest that concurrent expression of AR and RB was contributed for palbociclib-induced G1 arrest in TNBC cells.

### Palbociclib and enzalutamide do not result in apoptosis in TNBC cells

**Fig 5:**
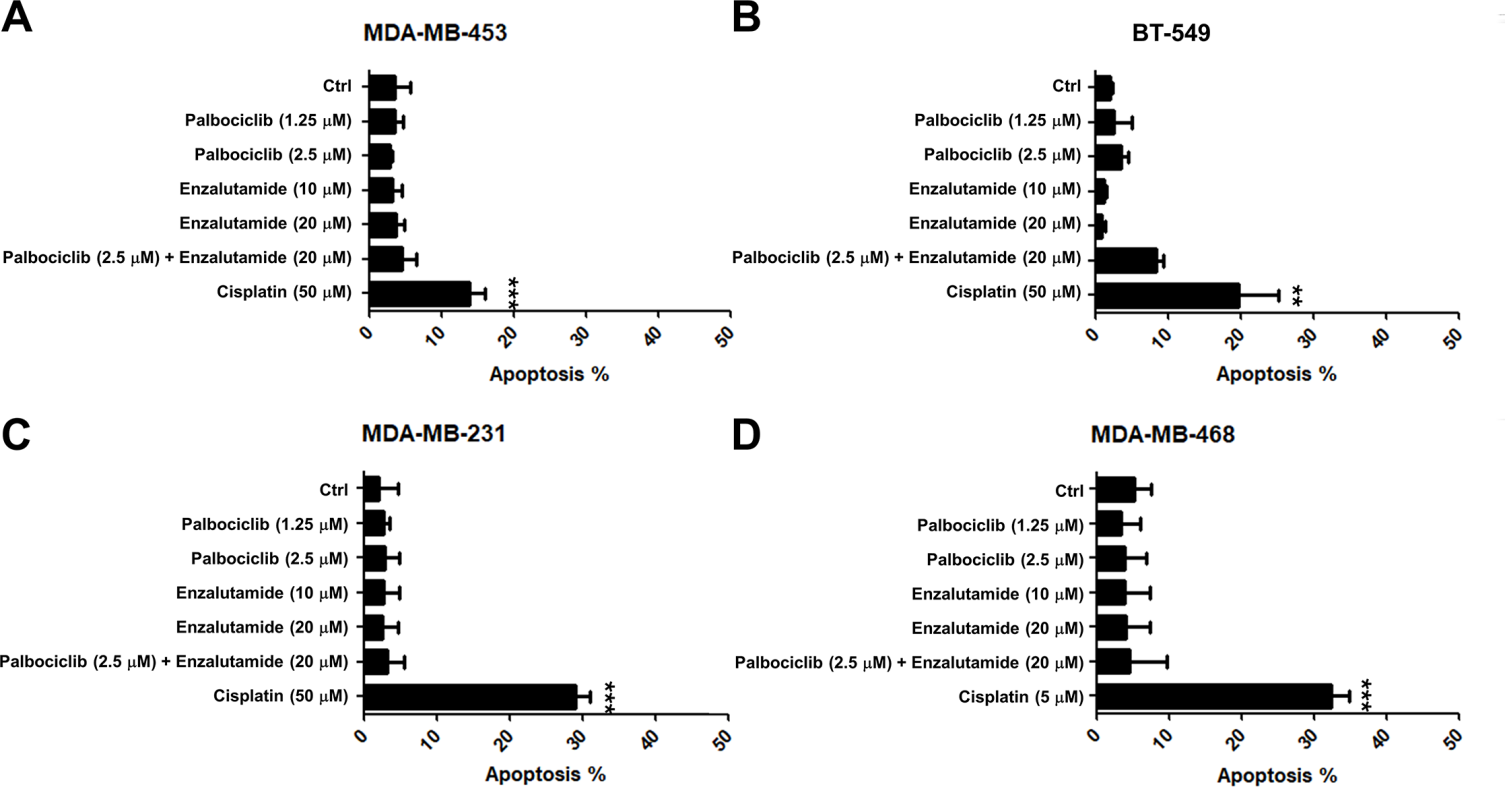
Palbociclib and enzalutamide do not result in apoptosis in TNBC cells. (A) MDA-MB-453, (B) BT-549, (C) MDA-MB-231 and (D) MDA-MB-468 cells were treated with DMSO (Ctrl), 1.25 and 2.5 μM palbociclib, 10 and 20 μM enzalutamide, combination of 2.5 μM palbociclib and 10 μM enzalutamide, cisplatin, and doxorubicin for 48 h, the percentage of apoptotic cells were analyzed by flow cytometry. The means ± SEM of three independent experiments performed in triplicate are shown. **P < 0.01; ***P < 0.001.

Due to the fact that palbociclib and enzalutamide have been reported to induce apoptosis in cancer, we further investigated the effect of palbociclib and enzalutamide on apoptosis in TNBC cells. We found that neither treatment alone nor combined treatment induced apoptosis, but cisplatin treatment increased the apoptotic cells in TNBC cell lines (Fig 5A–5D). These results indicated that the anti-cancer effect of palbociclib and enzalutamide was dependent on repression of cell growth rather than induction of apoptosis in TNBC cells.

### Clinical significances of *AR* and *RB1* gene expression in TNBC

**Fig 6:**
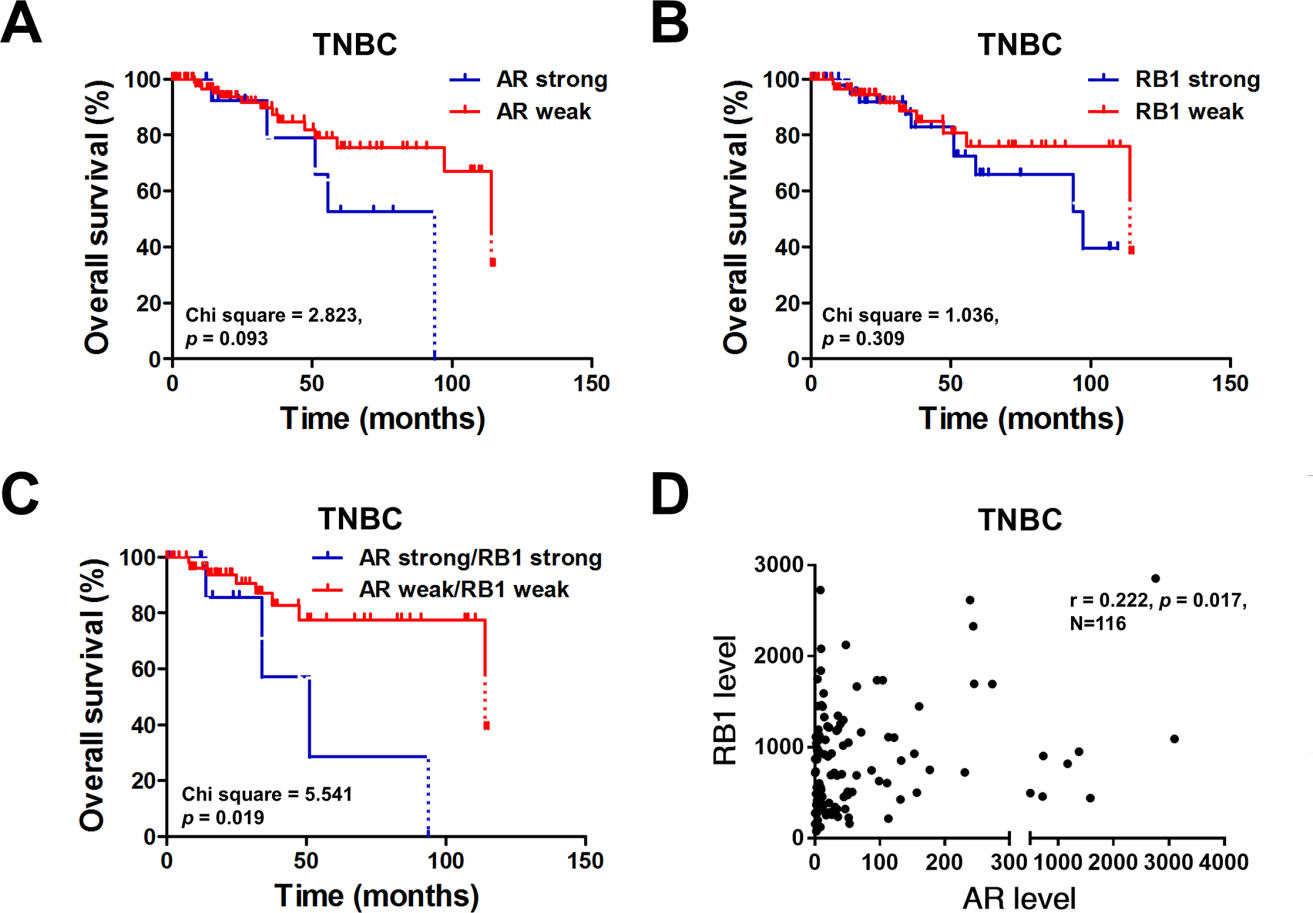
Clinical significances of *AR* and *RB1* gene expression in patients with TNBC. (A-D) The level 3 data of mRNA RSEM (RNA-Seq by Expectation Maximization) from patients with TNBC were selected and downloaded from the TCGA and Broad GDAC Firehose data portal. The mean of *AR* or *RB1* gene expressions were the chosen as cut-off value for separating tumors with strong and weak expression. Overall survival rates of TNBC patients were plotted against time in month for different parameters: the level of *AR* gene (A), the level of *RB1* gene (B), and the level of *AR*/*RB1* genes (C). The correlation between *AR* and *RB1* mRNA expressions was analyzed by Pearson correlation analysis (D).

To determine the clinical significances of *AR* and *RB1* gene expression in TNBC, we first examined the expression of AR in TNBC samples. We downloaded the tumor genome atlas (TCGA) data from cBioPortal website (http://www.cbioportal.org/) [15, 16], and analyzed the association between the levels of *AR* and *RB1* and clinical variables. The results showed that expression levels of *AR* or *RB1* did not link to overall survival of patients with TNBC (Fig 6A and 6B). However, we divided breast cancer patients into *AR*/*RB1* double-strong expression and *AR*/*RB1* double-weak expression cohorts. The results showed that TNBC patients with double-strong expression of *AR*/*RB1* significantly associated with poor overall survival (Fig 6C). The expression of AR was not correlated with age and tumor stage (S1 Table). Moreover, the expression of AR is correlated with RB1 in patients with TNBC (Fig 6D) and breast cancer (S4 Fig). In addition, the tissue array was analyzed by IHC, AR expression in TNBC samples is about 11.6% (S3 Fig) whereas the expression of AR was not associated with recurrence-free survival (S3 Fig). Results from tissue array were consistent with TCGA database. There results indicated that concurrent expression of AR and RB1 might be the biomarker in selecting patients who have therapeutic benefit from combining CDK4/6 inhibitors with AR antagonists.

## Discussion

In this study, we demonstrated that both AR and RB status were important for the response to palbociclib in TNBC cells. In addition, palbociclib in combination with enzalutamide enhanced the cytostatic effect in AR-positive/RB-proficient (MDA-MB-453) TNBC cells but not in AR-positive/RB-negative (BT-549), AR-negative/RB-proficient (MDA-MB-231) and AR-negative/RB-negative (MDA-MB-468) TNBC cells. Moreover, concurrent expression of AR and RB was contributed for palbociclib-induced G1 arrest in TNBC cells.

Enzalutamide, which is a second-generation AR antagonist, has a higher anticancer potency than first-generation AR inhibitors (bicalutamide and flutamide), including higher affinity for AR, repression of AR nuclear translocation, decrease in DNA binding and coactivator recruitment [17–19]. Enzalutamide has been approved by the U.S. Food and Drug Administration (FDA) for the treatment of patients with metastatic castration-resistant or chemo-resistant prostate cancer [10, 11, 20–22]. In addition to prostate cancer, growing evidences showed that enzalutamide also exerted anticancer effect on TNBC, which implied that targeting AR might be a good strategy for TNBC [12–14]. At least 3 clinical trials of neoadjuvant enzalutamide in combination with or without chemotherapy in TNBC patients are currently ongoing or just-completed (ClinicalTrials.gov Identifier: NCT02689427, NCT01889238 and NCT02457910). In our present study, we found that enzalutamide enhanced the palbociclib-induced cytostatic effect in AR-positive/RB-proficient (MDA-MB-453) cells rather than AR-positive/RB-negative (BT-549), AR-negative/RB-proficient (MDA-MB-231) and AR-negative/RB-negative (MDA-MB-468) cells (Fig 2). Our results provided a preclinical rationale in selecting patients who might have therapeutic benefit by combining CDK4/6 inhibitors with AR antagonists.

Palbociclib is a specific CDK4/6 inhibitor, which decreases the phosphorylation of RB and subsequently inhibits cancer cell growth [5, 6]. Although palbociclib was believed to exhibit anti-cancer potencies in an RB-dependent manner, increasing evidences showed that another potential biomarker was necessary for the response of RB-proficient cancer cells to palbociclib [23]. For example, either deletion of *CDKN2A* (p16^INK4A^) or low-expression of E2F transcription factor 1 (E2F1) was the significant predictor of the response to palbociclib in renal cell carcinoma (RCC) cell lines [24], and ER-positive breast cancer cell lines with high-expression of cyclin D1 were more sensitive to palbociclib [7]. Moreover, RB-proficient ovarian cancer cell lines with low-expression of p16^INK4A^ were more responsive to palbociclib [25]. Our data indicated that enzalutamide promoted palbociclib-induced G1 arrest in AR-positive/RB-proficient cells (Fig 3). RB is a transcriptional repressor which required for G1 to S phase transition. Previous studied demonstrated that RB interacts with AR in an androgen-independent manner and acts as a coactivator for AR [26]. In addition, AR could stimulate DNA replication via hyperphosphorylated RB indirectly in prostate cancer cells [27]. Palbociclib decreases the phosphorylation of RB, and enzalutamide might decrease the RB coactivator recruitment leading to RB-mediated cell cycle arrest.

TNBC with genetic loss of PR/ER/HER2-amplification and molecular heterogeneity has no approved target therapy to date [28–30]. Recent studies have shown that the molecular profiling in TNBC has revealed a number of potential targets for TNBC patients, including epidermal growth factor receptor (EGFR), mitogen-activated protein kinase (MAPK), proteasome subunits, and poly (ADP-ribose) polymerases (PARPs), Several compounds of these candidates have entered clinical trials for TNBC patients [31]. TNBC patients with double-strong expression of AR/RB1 significantly associated with poor overall survival (Fig 6C). Gucalp *et al*. evaluated AR status using IHC in 424 patients with TNBC, 12% AR positive with > 10% nuclear staining [32]. The level of RB1 mRNA correlated with level of pRB [33] and RB is reported that lost in ~40% of TNBC [34]. Furthermore, pRB level was found that lost in most TNBC by IHC analysis [35]. Studies have identified that luminal androgen receptor (LAR) subtype of TNBC cell lines might be sensitive to CDK4/6 inhibition [36, 37]. Asghar *et al*. demonstrated that MFM223 and SUM185 cells were sensitive to palbociclib [37]. Indeed, these two LAR subtype cell lines harbored high level of pRB. The xenografts of LAR MDA-MB-453 cells were significantly reduced by palbociclib treatment [37]. These findings supported AR positive and RB proficient TNBC were suitable for palbociclib treatment. In fact, a clinical trial of palbociclib in combination with bicalutamide, a non-steroidal AR inhibitor, for the treatment of patients with AR-positive TNBC is ongoing (ClinicalTrials.gov Identifier: NCT02605486). In the present study, palbociclib plus enzalutamide was found to effectively repress AR-positive/RB-proficient TNBC cell growth suggesting that co-expression of AR and RB might be a biomarker for combined treatment of palbociclib with enzalutamide in TNBC.

Current study has some limitation, first of all only four TNBC cell lines were used and there might be other protein profile or mutants that can contribute to the differential effects of palbociclib and enzalutamide and combination in these cell lines. Furthermore, despite we showed that knockdown RB attenuated palbociclib-mediated effect on AR+/RB+ MDA-MB-453 cells, the notion that combination effect of palbociclib and enzalutamide is most significant in AR+/RB+ MDA-MB-453 cells needs further mechanistic studies to validate that AR is essential for palbociclib-mediated cell cycle arrest in AR+/RB+ TNBC cells. Last but not the least; our study is limited by lack of sufficient and consolidative clinical data to support the possible association between RB and AR. It is also not clear and inconclusive with regards to the role of AR and RB expressions in TNBC tumors. More clinical studies are needed to address or validate the biological association between AR and RB.

In conclusion, we found that palbociclib effectively inhibited RB-proficient TNBC cell growth and the expression of AR might contribute for palbociclib-mediated G1 arrest. Moreover, enzalutamide enhanced the palbociclib-induced cytostatic effect in AR-positive/RB-proficient TNBC cells indicating that palbociclib in combination with enzalutamide may be a therapeutic strategy for AR-positive/RB-proficient TNBCs.

## Supporting information

S1 Table The relationship*s* between *AR* gene expression and clinical variables. The level 3 data of mRNA RSEM in patients with TNBC were downloaded from the TCGA and Broad GDAC Firehose data portal. The mean of *AR* or *RB1* gene expressions were the chosen as cut-off value for separating tumors with strong and weak expression which were analyzed for relationship between *AR* gene expression and clinical variables.
(DOCX)

S1 Fig The effects of palbociclib and enzalutamide treatment in MCF 10A cells. (A) MCF 10A cells were treated with various concentrations of palbociclib, (B) enzalutamide and (C) combination of 2.5 μM palbociclib and 20 μM enzalutamide for 72 h, the cell viability was determined using MTT assay. The means ± SEM of three independent experiments performed in triplicate are shown.
(TIF)

S2 Fig The effects of palbociclib are attenuated by RB knockdown. MDA-MB-453 cells were transfected with siRNA against control (siCtrl) and RB1 (siRB) for 24 h, and the transfected cells were further treated with 2.5 μM palbociclib for 48 h. The treated cells were analyzed by flow cytometry analysis.
(TIF)

S3 Fig AR expression could be detected in patients with TNBC and did not link to recurrence-free survival. (A) Representative tissue microarray of immunohistochemical expression of AR in TNBC samples and the events of AR expression. (B) recurrence-free survival of TNBC patients were plotted against time in month for the level of *AR* gene.
(TIF)

S4 Fig The correlation between *AR* and *RB1* expression in patients with breast cancer. The level 3 data of mRNA RSEM in breast cancer were downloaded from the TCGA and Broad GDAC Firehose data portal. The correlation between *AR* and *RB1* mRNA was analyzed by Pearson correlation analysis.
(TIF)

## References

[1] Gelmon K, Dent R, Mackey JR, Laing K, McLeod D, Verma S. Targeting triple-negative breast cancer: optimising therapeutic outcomes. Ann Oncol. 2012;23(9):2223–34. doi: 10.1093/annonc/mds067 22517820 10.1093/annonc/mds067

[2] Kobayashi K, Ito Y, Matsuura M, Fukada I, Horii R, Takahashi S, et al Impact of immunohistological subtypes on the long-term prognosis of patients with metastatic breast cancer. Surg Today. 2016;46(7):821–6. doi: 10.1007/s00595-015-1252-x 26467559 10.1007/s00595-015-1252-x

[3] Malorni L, Shetty PB, De Angelis C, Hilsenbeck S, Rimawi MF, Elledge R, et al Clinical and biologic features of triple-negative breast cancers in a large cohort of patients with long-term follow-up. Breast Cancer Res Treat. 2012;136(3):795–804. doi: 10.1007/s10549-012-2315-y 23124476 10.1007/s10549-012-2315-y PMC3513514

[4] Mayer IA, Abramson VG, Lehmann BD, Pietenpol JA. New strategies for triple-negative breast cancer—deciphering the heterogeneity. Clin Cancer Res. 2014;20(4):782–90. doi: 10.1158/1078-0432.CCR-13-0583 24536073 10.1158/1078-0432.CCR-13-0583 PMC3962777

[5] Toogood PL, Harvey PJ, Repine JT, Sheehan DJ, VanderWel SN, Zhou H, et al Discovery of a potent and selective inhibitor of cyclin-dependent kinase 4/6. J Med Chem. 2005;48(7):2388–406. doi: 10.1021/jm049354h 15801831 10.1021/jm049354h

[6] Fry DW, Harvey PJ, Keller PR, Elliott WL, Meade M, Trachet E, et al Specific inhibition of cyclin-dependent kinase 4/6 by PD 0332991 and associated antitumor activity in human tumor xenografts. Mol Cancer Ther. 2004;3(11):1427–38. 15542782

[7] Finn RS, Dering J, Conklin D, Kalous O, Cohen DJ, Desai AJ, et al PD 0332991, a selective cyclin D kinase 4/6 inhibitor, preferentially inhibits proliferation of luminal estrogen receptor-positive human breast cancer cell lines in vitro. Breast Cancer Res. 2009;11(5):R77 doi: 10.1186/bcr2419 19874578 10.1186/bcr2419 PMC2790859

[8] Finn RS, Martin M, Rugo HS, Jones S, Im SA, Gelmon K, et al Palbociclib and Letrozole in Advanced Breast Cancer. N Engl J Med. 2016;375(20):1925–36. doi: 10.1056/NEJMoa1607303 27959613 10.1056/NEJMoa1607303

[9] Turner NC, Ro J, Andre F, Loi S, Verma S, Iwata H, et al Palbociclib in Hormone-Receptor-Positive Advanced Breast Cancer. N Engl J Med. 2015;373(3):209–19. doi: 10.1056/NEJMoa1505270 26030518 10.1056/NEJMoa1505270

[10] Scher HI, Fizazi K, Saad F, Taplin ME, Sternberg CN, Miller K, et al Increased survival with enzalutamide in prostate cancer after chemotherapy. N Engl J Med. 2012;367(13):1187–97. doi: 10.1056/NEJMoa1207506 22894553 10.1056/NEJMoa1207506

[11] Beer TM, Tombal B. Enzalutamide in metastatic prostate cancer before chemotherapy. N Engl J Med. 2014;371(18):1755–6. doi: 10.1056/NEJMc1410239 25354111 10.1056/NEJMc1410239

[12] Caiazza F, Murray A, Madden SF, Synnott NC, Ryan EJ, O'Donovan N, et al Preclinical evaluation of the AR inhibitor enzalutamide in triple-negative breast cancer cells. Endocr Relat Cancer. 2016;23(4):323–34. doi: 10.1530/ERC-16-0068 26932782 10.1530/ERC-16-0068

[13] Barton VN, D'Amato NC, Gordon MA, Lind HT, Spoelstra NS, Babbs BL, et al Multiple molecular subtypes of triple-negative breast cancer critically rely on androgen receptor and respond to enzalutamide in vivo. Mol Cancer Ther. 2015;14(3):769–78. doi: 10.1158/1535-7163.MCT-14-0926 25713333 10.1158/1535-7163.MCT-14-0926 PMC4534304

[14] Cochrane DR, Bernales S, Jacobsen BM, Cittelly DM, Howe EN, D'Amato NC, et al Role of the androgen receptor in breast cancer and preclinical analysis of enzalutamide. Breast Cancer Res. 2014;16(1):R7 doi: 10.1186/bcr3599 24451109 10.1186/bcr3599 PMC3978822

[15] Cerami E, Gao J, Dogrusoz U, Gross BE, Sumer SO, Aksoy BA, et al The cBio cancer genomics portal: an open platform for exploring multidimensional cancer genomics data. Cancer Discov. 2012;2(5):401–4. doi: 10.1158/2159-8290.CD-12-0095 22588877 10.1158/2159-8290.CD-12-0095 PMC3956037

[16] Gao J, Aksoy BA, Dogrusoz U, Dresdner G, Gross B, Sumer SO, et al Integrative analysis of complex cancer genomics and clinical profiles using the cBioPortal. Sci Signal. 2013;6(269):pl1 doi: 10.1126/scisignal.2004088 23550210 10.1126/scisignal.2004088 PMC4160307

[17] Farrow JM, Yang JC, Evans CP. Autophagy as a modulator and target in prostate cancer. Nat Rev Urol. 2014;11(9):508–16. doi: 10.1038/nrurol.2014.196 25134829 10.1038/nrurol.2014.196 PMC4415606

[18] Loriot Y, Bianchini D, Ileana E, Sandhu S, Patrikidou A, Pezaro C, et al Antitumour activity of abiraterone acetate against metastatic castration-resistant prostate cancer progressing after docetaxel and enzalutamide (MDV3100). Ann Oncol. 2013;24(7):1807–12. doi: 10.1093/annonc/mdt136 23576708 10.1093/annonc/mdt136

[19] Tran C, Ouk S, Clegg NJ, Chen Y, Watson PA, Arora V, et al Development of a second-generation antiandrogen for treatment of advanced prostate cancer. Science. 2009;324(5928):787–90. doi: 10.1126/science.1168175 19359544 10.1126/science.1168175 PMC2981508

[20] Scher HI, Beer TM, Higano CS, Anand A, Taplin ME, Efstathiou E, et al Antitumour activity of MDV3100 in castration-resistant prostate cancer: a phase 1–2 study. Lancet. 2010;375(9724):1437–46. doi: 10.1016/S0140-6736(10)60172-9 20398925 10.1016/S0140-6736(10)60172-9 PMC2948179

[21] Schrader AJ, Boegemann M, Ohlmann CH, Schnoeller TJ, Krabbe LM, Hajili T, et al Enzalutamide in castration-resistant prostate cancer patients progressing after docetaxel and abiraterone. Eur Urol. 2014;65(1):30–6. doi: 10.1016/j.eururo.2013.06.042 23849416 10.1016/j.eururo.2013.06.042

[22] Loriot Y, Miller K, Sternberg CN, Fizazi K, De Bono JS, Chowdhury S, et al Effect of enzalutamide on health-related quality of life, pain, and skeletal-related events in asymptomatic and minimally symptomatic, chemotherapy-naive patients with metastatic castration-resistant prostate cancer (PREVAIL): results from a randomised, phase 3 trial. Lancet Oncol. 2015;16(5):509–21. doi: 10.1016/S1470-2045(15)70113-0 25888263 10.1016/S1470-2045(15)70113-0

[23] O'Leary B, Finn RS, Turner NC. Treating cancer with selective CDK4/6 inhibitors. Nat Rev Clin Oncol. 2016;13(7):417–30. doi: 10.1038/nrclinonc.2016.26 27030077 10.1038/nrclinonc.2016.26

[24] Logan JE, Mostofizadeh N, Desai AJ, VONE E, Conklin D, Konkankit V, et al PD-0332991, a potent and selective inhibitor of cyclin-dependent kinase 4/6, demonstrates inhibition of proliferation in renal cell carcinoma at nanomolar concentrations and molecular markers predict for sensitivity. Anticancer Res. 2013;33(8):2997–3004. 23898052

[25] Konecny GE, Winterhoff B, Kolarova T, Qi J, Manivong K, Dering J, et al Expression of p16 and retinoblastoma determines response to CDK4/6 inhibition in ovarian cancer. Clin Cancer Res. 2011;17(6):1591–602. doi: 10.1158/1078-0432.CCR-10-2307 21278246 10.1158/1078-0432.CCR-10-2307 PMC4598646

[26] Yeh S, Miyamoto H, Nishimura K, Kang H, Ludlow J, Hsiao P, et al Retinoblastoma, a tumor suppressor, is a coactivator for the androgen receptor in human prostate cancer DU145 cells. Biochem Biophys Res Commun. 1998;248(2):361–7. doi: 10.1006/bbrc.1998.8974 9675141 10.1006/bbrc.1998.8974

[27] Gao S, Gao Y, He HH, Han D, Han W, Avery A, et al Androgen Receptor Tumor Suppressor Function Is Mediated by Recruitment of Retinoblastoma Protein. Cell Rep. 2016;17(4):966–76. doi: 10.1016/j.celrep.2016.09.064 27760327 10.1016/j.celrep.2016.09.064 PMC5123835

[28] Lehmann BD, Bauer JA, Chen X, Sanders ME, Chakravarthy AB, Shyr Y, et al Identification of human triple-negative breast cancer subtypes and preclinical models for selection of targeted therapies. J Clin Invest. 2011;121(7):2750–67. doi: 10.1172/JCI45014 21633166 10.1172/JCI45014 PMC3127435

[29] Lehmann BD, Pietenpol JA. Identification and use of biomarkers in treatment strategies for triple-negative breast cancer subtypes. J Pathol. 2014;232(2):142–50. doi: 10.1002/path.4280 24114677 10.1002/path.4280 PMC4090031

[30] Prat A, Adamo B, Cheang MC, Anders CK, Carey LA, Perou CM. Molecular characterization of basal-like and non-basal-like triple-negative breast cancer. Oncologist. 2013;18(2):123–33. doi: 10.1634/theoncologist.2012-0397 23404817 10.1634/theoncologist.2012-0397 PMC3579595

[31] Kalimutho M, Parsons K, Mittal D, Lopez JA, Srihari S, Khanna KK. Targeted Therapies for Triple-Negative Breast Cancer: Combating a Stubborn Disease. Trends Pharmacol Sci. 2015;36(12):822–46. doi: 10.1016/j.tips.2015.08.009 26538316 10.1016/j.tips.2015.08.009

[32] Gucalp A, Tolaney S, Isakoff SJ, Ingle JN, Liu MC, Carey LA, et al Phase II trial of bicalutamide in patients with androgen receptor-positive, estrogen receptor-negative metastatic Breast Cancer. Clin Cancer Res. 2013;19(19):5505–12. doi: 10.1158/1078-0432.CCR-12-3327 23965901 10.1158/1078-0432.CCR-12-3327 PMC4086643

[33] Subhawong AP, Subhawong T, Nassar H, Kouprina N, Begum S, Vang R, et al Most basal-like breast carcinomas demonstrate the same Rb-/p16+ immunophenotype as the HPV-related poorly differentiated squamous cell carcinomas which they resemble morphologically. Am J Surg Pathol. 2009;33(2):163–75. doi: 10.1097/PAS.0b013e31817f9790 18936692 10.1097/PAS.0b013e31817f9790 PMC2965595

[34] Witkiewicz AK, Knudsen ES. Retinoblastoma tumor suppressor pathway in breast cancer: prognosis, precision medicine, and therapeutic interventions. Breast Cancer Res. 2014;16(3):207 doi: 10.1186/bcr3652 25223380 10.1186/bcr3652 PMC4076637

[35] Robinson TJ, Liu JC, Vizeacoumar F, Sun T, Maclean N, Egan SE, et al RB1 status in triple negative breast cancer cells dictates response to radiation treatment and selective therapeutic drugs. PLoS One. 2013;8(11):e78641 doi: 10.1371/journal.pone.0078641 24265703 10.1371/journal.pone.0078641 PMC3827056

[36] Asghar U, Herrera-Abreu MT, Cutts R, Babina I, Pearson A, Turner NC. Identification of subtypes of triple negative breast cancer (TNBC) that are sensitive to CDK4/6 inhibition. Journal of Clinical Oncology. 2015;33(15_suppl):11098–. doi: 10.1200/jco.2015.33.15_suppl.11098

[37] Asghar US, Barr AR, Cutts R, Beaney M, Babina I, Sampath D, et al Single-Cell Dynamics Determines Response to CDK4/6 Inhibition in Triple-Negative Breast Cancer. Clin Cancer Res. 2017;23(18):5561–72. doi: 10.1158/1078-0432.CCR-17-0369 28606920 10.1158/1078-0432.CCR-17-0369 PMC6175044

