# Supplementary material for: Combination of palbociclib with enzalutamide shows in vitro activity in RB proficient and androgen receptor positive triple negative breast cancer cells
Source: PLoS One. 2017 Dec 20;12(12):e0189007. doi: 10.1371/journal.pone.0189007 (PMC5737960; doi:10.1371/journal.pone.0189007)
Supplement: S1 Table — The level 3 data of mRNA RSEM in patients with TNBC were downloaded from the TCGA and Broad GDAC Firehose data portal. The mean of AR or RB1 gene expressions were the chosen as cut-off value for separating tumors with strong and weak expression which were analyzed for relationship between AR gene expression and clinical variables. (DOCX) [file pone.0189007.s001.docx]

**S1 Table. The relationship*s* between *AR* gene expression and clinical variables.**

|  |  | AR expression | | | |  |
| --- | --- | --- | --- | --- | --- | --- |
|  |  | weak | % | strong | % | *p* value |
| Age (year) | <65 | 79 | 79.80 | 16 | 94.12 | 0.138 |
|  | ≥65 | 20 | 20.20 | 1 | 5.88 |  |
| Stage AJCC VI | 1 | 17 | 17.17 | 2 | 11.76 | 0.384 |
|  | 2 | 64 | 64.65 | 9 | 52.94 |  |
|  | 3 | 14 | 14.14 | 5 | 29.41 |  |
|  | 4 | 2 | 2.02 | 0 | 0.00 |  |
|  | N/A | 2 | 2.02 | 1 | 5.88 |  |

The level 3 data of mRNA RSEM in patients with TNBC were downloaded from the TCGA and Broad GDAC Firehose data portal. The mean of *AR* or *RB1* gene expressions were the chosen as cut-off value for separating tumors with strong and weak expression which were analyzed for relationship between *AR* gene expression and clinical variables.
