# Supplementary figures and images for: Combination of palbociclib with enzalutamide shows in vitro activity in RB proficient and androgen receptor positive triple negative breast cancer cells
Source: PLoS One. 2017 Dec 20;12(12):e0189007. doi: 10.1371/journal.pone.0189007 (PMC5737960; doi:10.1371/journal.pone.0189007)

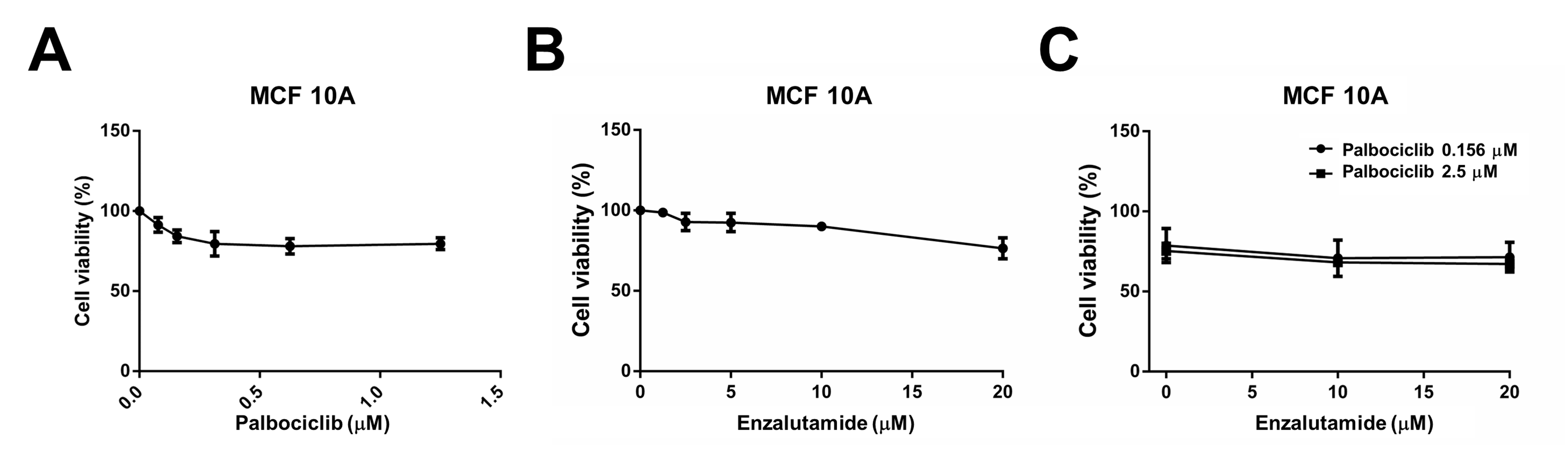

Supplement: S1 Fig — (A) MCF 10A cells were treated with various concentrations of palbociclib, (B) enzalutamide and (C) combination of 2.5 μM palbociclib and 20 μM enzalutamide for 72 h, the cell viability was determined using MTT assay. The means ± SEM of three independent experiments performed in triplicate are shown. (TIF) [file pone.0189007.s002.TIF]

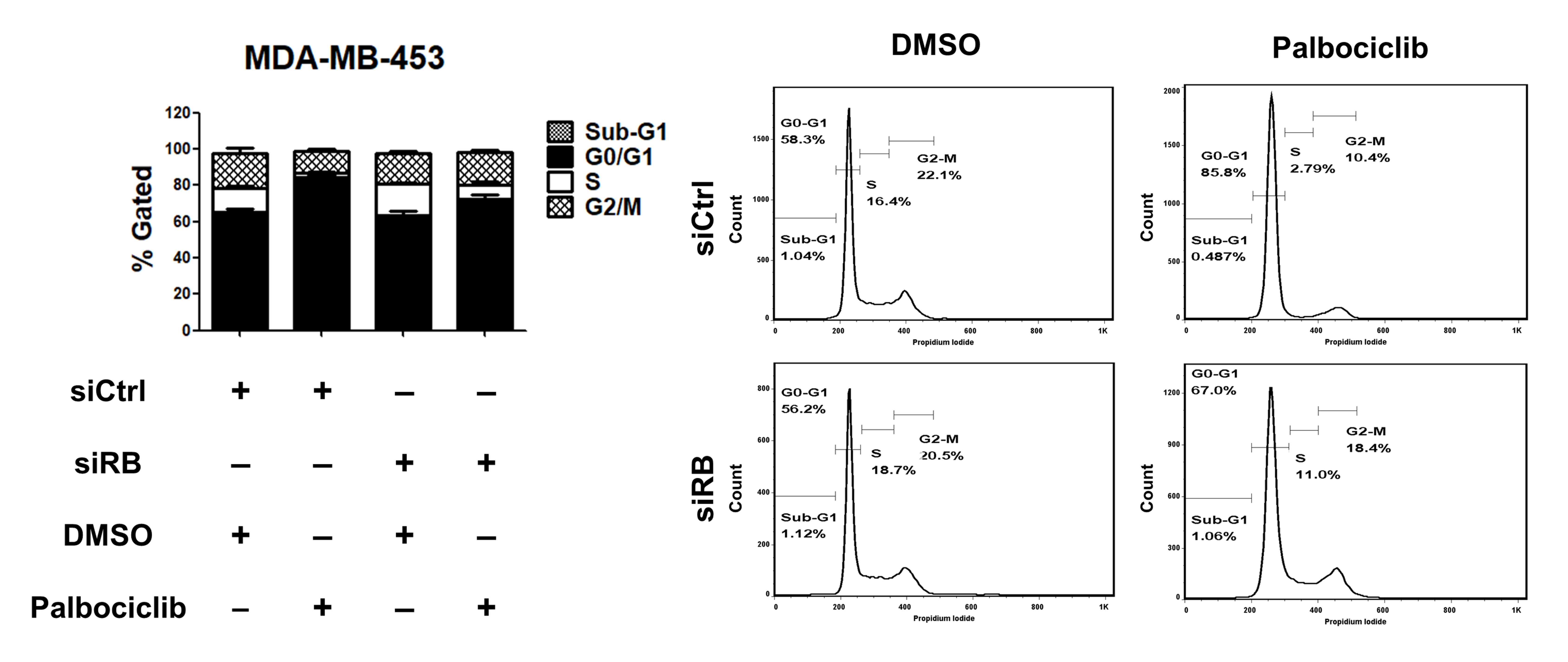

Supplement: S2 Fig — MDA-MB-453 cells were transfected with siRNA against control (siCtrl) and RB1 (siRB) for 24 h, and the transfected cells were further treated with 2.5 μM palbociclib for 48 h. The treated cells were analyzed by flow cytometry analysis. (TIF) [file pone.0189007.s003.TIF]

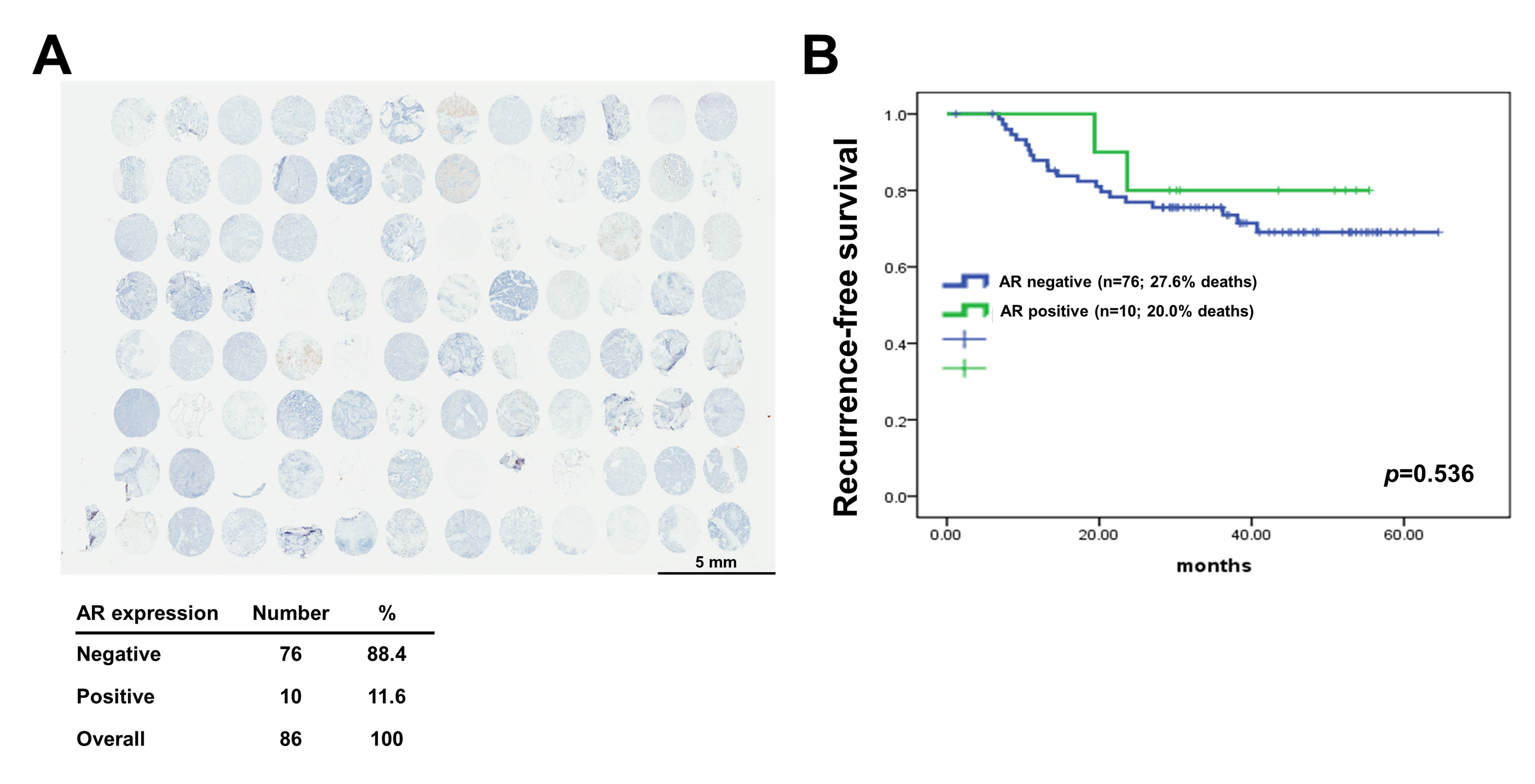

Supplement: S3 Fig — (A) Representative tissue microarray of immunohistochemical expression of AR in TNBC samples and the events of AR expression. (B) recurrence-free survival of TNBC patients were plotted against time in month for the level of AR gene. (TIF) [file pone.0189007.s004.TIF]

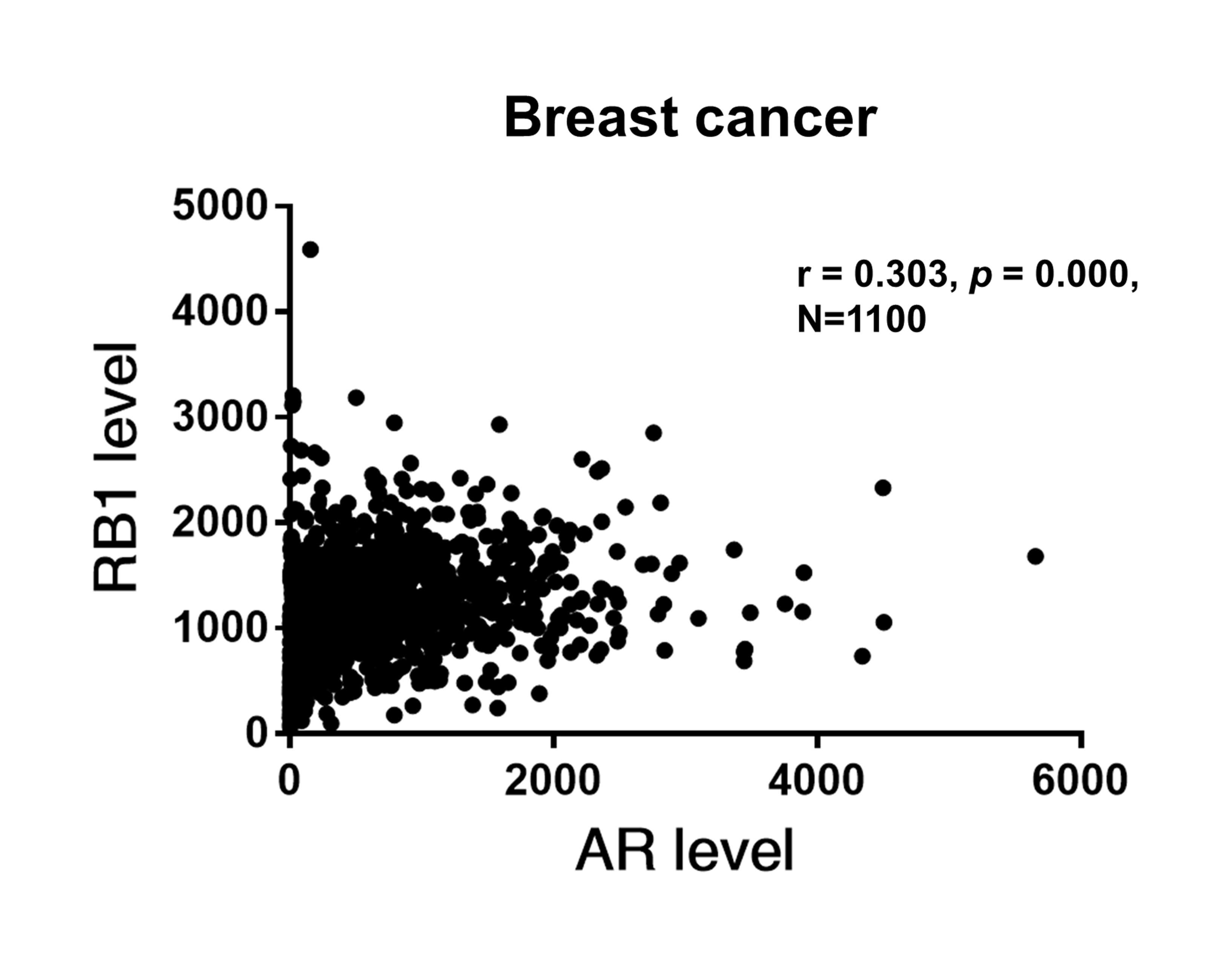

Supplement: S4 Fig — The level 3 data of mRNA RSEM in breast cancer were downloaded from the TCGA and Broad GDAC Firehose data portal. The correlation between AR and RB1 mRNA was analyzed by Pearson correlation analysis. (TIF) [file pone.0189007.s005.TIF]
